# Nanotherapeutics Using an HIV-1 Poly A and Transactivator of the HIV-1 LTR-(TAR-) Specific siRNA

**DOI:** 10.4061/2011/719139

**Published:** 2011-05-10

**Authors:** Supriya D. Mahajan, Ravikumar Aalinkeel, Jessica L. Reynolds, Bindukumar Nair, Donald E. Sykes, Wing-Cheung Law, Hong Ding, Earl J. Bergey, Paras N. Prasad, Stanley A. Schwartz

**Affiliations:** ^1^Division of Allergy, Immunology, and Rheumatology, Department of Medicine, University at Buffalo, The State University of New York, 640 Ellicott Street, Room 444 Innovation Center, Buffalo, NY 14203, USA; ^2^Institute for Lasers, Photonics and Biophotonics, University at Buffalo, The State University of New York, Buffalo, NY 14260, USA

## Abstract

HIV-1 replication can be efficiently inhibited by intracellular expression of an siRNA targeting the viral RNA. We used a well-validated siRNA (si510) which targets the poly A/TAR (transactivator of the HIV-1 LTR) site and suppresses viral replication. Nanotechnology holds much potential for impact in the field of HIV-1 therapeutics, and nanoparticles such as quantum rods (QRs) can be easily functionalized to incorporate siRNA forming stable nanoplexes that can be used for gene silencing. We evaluated the efficacy of the QR-si510 HIV-1 siRNA nanoplex in suppressing viral replication in the HIV-1-infected monocytic cell line THP-1 by measuring p24 antigen levels and gene expression levels of HIV-1 LTR. Our results suggest that the QR-si510 HIV-1 siRNA nanoplex is not only effective in delivering siRNA, but also in suppressing HIV-1 viral replication for a longer time period. HIV-1 nanotherapeutics can thus enhance systemic bioavailability and offer multifunctionality.

## 1. Introduction

Treatment of human immunodeficiency virus type 1 (HIV-1) disease with highly active antiretroviral therapy (HAART) has enormous clinical benefits; however, there exists the problem of continuing emergence of drug resistance, treatment adherence and serious risk of toxicities, with the prospect of life-long antiretroviral treatment, additionally viral reservoirs persist in several sequestered sites such as the central nervous system (CNS) and testis. Therefore, developing strategies that treat HIV infection and eliminate persistent viral reservoirs are needed and gene therapy approaches such as RNA interference (RNAi) have emerged as a therapeutic possibility for the elimination of HIV-1 [1–3]. 

RNAi works either through binding with HIV-1 to inhibit provirus integration into cellular genome or with mRNA products to inhibit genes that play a vital role in HIV-1 infectivity by knocking down the virulence capacity of the virus [2, 3]. RNAi is a highly potent mechanism of posttranscriptional gene silencing which is mediated by sequence-specific siRNAs, which can effectively down regulate expression of either a cellular RNA target molecules by selective degradation of mRNAs [4–6]. Viral replication can be successfully inhibited using siRNA; however, designing a functional siRNA that targets a highly divergent virus can be challenging [1–5]. Recently, a very elegant and comprehensive study by Naito et al. analyzed several promising siRNA sequences of near full length HIV-1 group M strains from the Los Alamos sequence database and selected a set of optimal antiviral siRNA [7, 8]. Delivery of optimal amounts of these synthetic antiviral siRNAs by transfection into cultured cells can effectively inhibit HIV-1 infections; however, the antiHIV-1 effects of these delivery methods are transient due to the degradation and dilution of siRNAs during cell division.

 A major hurdle limiting the use of the gene silencing technology is the lack of methods to safely and efficiently deliver siRNA molecules to target cells/tissues. In the free form, siRNA molecules have a very short half life in physiological conditions, owing to their vulnerability for degradation by endogeneous nucleases. Therefore, they need macromolecular carriers (vectors) that will not only protect them from degradation in the biological milieu, but also steer them to desired cells/tissues as well as facilitate their cellular entry. Nanoparticle surfaces can be easily modified to incorporate cationic charges, which will facilitate their stable electrostatic complexation with anionic genetic materials such as siRNA, for the purpose of targeted gene delivery/silencing [9–11]. HIV-1 nanotherapeutics can increase drug solubility, enhance systemic bioavailability, and at the same time offer multifunctionality. 

 In the current study, we have used a synthetic siRNA (si510) described by Naito et al. [7, 8] which is a highly effective siRNA against divergent HIV-1 strains which targets the poly A/TAR (transactivator of the HIV-1 LTR) site and successfully targets viral transcripts and achieves effective viral inhibition. HIV-1 TAR RNA is the binding site of the viral protein Tat, the transactivator of the HIV-1 LTR (long terminal repeat). It is present at the 5′ end of all HIV-1 spliced and unspliced mRNAs in the nucleus as well as in the cytoplasm. A TAR-specific RNA will affect the viral and cellular binding factors thereby decreasing virion production and effectively inhibit viral replication [12]. HIV-1 contains duplicated long terminal repeat (LTR) sequences flanking the proviral genome. These LTRs contain identical poly(A) (polyadenylation) signals, which are both transcribed into RNA, to allow efficient viral expression [12]. Thus the si510 siRNA that targets both the poly A/TAR can effectively suppress viral replication. Naito et al. reported that the siRNA transfections using the si510 sequence that were done in their study were done using a commercially available reagent Lipofectamine (Invitrogen, CA) and that this transfection was transient in nature and that the percentage decrease in viral replication was >90%, 48 hours posttransfection [7]. The goal of the current investigation to evaluate if complexing the si510 siRNA to a nanoparticle such as a quantum rod (QR) would effectively suppress viral replication for a extended period of time. In addition to efficacy, the dose and time kinetics of this siRNA nanoplex was evaluated in the HIV-1-infected monocytic cell line THP-1. We believe that the results of this study will attest to the fact that nanotechnology can play a pivotal role in HIV-1 therapeutics.

## 2. Methods

### 2.1. Study Design

Following synthesis and characterization of the nanoplexes (QR-si510 siRNA complex), we evaluated their uptake in vitro cultured monolayers of an HIV-1-infected monocytic cell line THP-1 using confocal imaging. THP-1 cells were treated with various concentrations of the si510 siRNA ranging from 5 nM to 100 nM over a range of time periods from 6 hr to 1 week posttransfection and the antiviral efficacy the nanoplexes was tested. Two independent methods were used to evaluate antiviral effects: (1) the measurement of p24 antigen levels using a commercially available p24 ELISA assay and (2) quantitating HIV-1 LTR-R/U5 gene expression using quantitative real-time PCR.

### 2.2. Synthesis and Aqueous Dispersion of the QRs Terminated with Carboxyl Groups

The formation of the Cadmium (Cd) Selelinum (Se) CdSe core nanocrystals, and thin layer of Zinc Sulfide (ZnS) shell (<1.5 nm) is grown over the nanocrystal core. CdS/ZnS graded shell on CdSe QRs in organic media was carried out as described by Yong et al. [13]. To form the nanocrystal core, 6 mmol cadmium oxide (CdO) was dissolved in 10 mL of oleic acid at 280°C, producing a homogeneous reaction mixture of cadmium complex. Then, a solution of selenium (Se) precursor was injected into the reaction mixture. The Se : Cd molar ratio was 1 : 3. The length of reaction time following the injection determines the final size of the nanocrystals. The CdSe nanocrystals were then purified by precipitation from the reaction mixture with ethanol followed by centrifugation. For the growth of a ZnS shell over the CdSe cores, the CdSe cores were dispersed in 5 g of TOPO at 250°C. A mixture of diethylzinc (Et_2_Zn) and hexamethyldisilathiane ((TMS)_2_S) (Zn : S = 1 : 1 molar ratio) were mixed with Trioctylphosphine (TOP) and the solution precursor was added dropwise into the CdSe core nanocrystals reaction mixture at 250°C. After ~20 minutes of heating, the heating mantle was removed, and then the reaction mixture was air cooled to room temperature. Following their synthesis, the CdSe/CdS/ZnS QRs were dispersed in aqueous media by coating with mercaptosuccinic acid (MSA). Briefly, 3 mmol of MSA was dissolved in 10 mL of chloroform under vigorous stirring. After stirring for 10 to 15 minutes, 2 mL of concentrated (~40 mg/mL chloroform) CdSe/CdS/ZnS QR dispersion was added into this mixture. This solution was stirred overnight at room temperature. The QRs were separated from the surfactant solution by addition of ethanol and centrifugation. The red-brownish precipitate was redispersed in 10 mL HPLC water and the solution was further filtered using a syringe filter with a pore diameter of 0.45 *μ*m. The QRs have relatively good colloidal stability and no precipitation was observed after several months of storage [13–15].

### 2.3. Physical Characterization of QRs

The physical properties of the nanoplex were established using transmission electron microscopy (TEM), dynamic light scattering (DLS), spectrophotometry and spectrofluorimetry. TEM was used to determine the particle size and size distribution. The aqueous dispersion of the nanoparticles was drop cast on a TEM copper grid (electron microscopy sciences, Inc.) and visualized using a JEOL JEM 2020 electron microscope. DLS measurements were carried out using a Brookhaven 90Plus particle size analyzer, with a scattering angle of 90°. UV-vis absorption spectra were recorded using a Shimadzu UV-3101 PC spectrophotometer, using a quartz cuvette with 1 cm path length. Fluorescence spectra were recorded on a Fluorolog-3 (Jobin Yvon, Longjumeau, France) spectrofluorimeter.

### 2.4. Cell Viability Measurement Using an MTT (3-(4,5-Dimethylthiazol-2-yl)-2,5-Diphenyltetrazolium Bromide, a Tetrazole) Assay

MTT cell proliferation assay measures the reduction of a tetrazolium component (MTT) into an insoluble formazan product by the mitochondria of viable cells. The MTT assay is a quantitative, sensitive detection of cell proliferation since it measures the growth rate of cells by virtue of a linear relationship between cell activity and absorbance. Typically 10,000 cells suspended in 100 *μ*L of media were incubated with 10 *μ*L of MTT reagent (Cat # 30-1010 K; ATCC) for approximately 3 hours, followed by addition of a detergent solution to lyse the cells and solubilize the colored crystals. Colorimetric detection was done at a wavelength of 570 nm. The amount of color produced is directly proportional to the number of viable cells.

### 2.5. Determination of the Delivery of Nanoplexes into THP-1 Cells

Nanoplexes (QD-siRNA) are discrete spheres, having a mean size of 15–20 nm. They are sufficiently small to diffuse into cells by endocytosis and favor nucleic acid transfer and release from the endosome across the nuclear membrane. Nanoplex suspensions are prepared by adding 5–100 nM si510 siRNA/10 ul volume to 20 *μ*L of QR solution suspended in a total of 200 *μ*L of serum-free media to permit complex formation for 15 min at room temperature followed by addition of the nanoplex to the THP-1 cells plated in 35 mm glass bottom culture plates. Commercial transfection reagent, Lipofectamine (Invitrogen, Carlsbad, CA) is used as a control and used according to the manufacturer's protocol. Cells are cultured in an incubator (VWR Scientific, model 2400) at 37°C in a humidified atmosphere of 5% CO_2_ for a period ranging from 12 hr to 1 week posttransfection.

### 2.6. Imaging of Nanoparticles

A Nikon Eclipse TE2000 microscope equipped with the Nuance GNIR imaging system (Cambridge Research & Instrumentation Inc., Cambridge, MA) was employed for cell imaging. This imaging system is capable of multispectral (wavelength-resolved) imaging in the range of 500–950 nm. Custom-designed filter cubes, with corresponding dichroic and emission filters acquired from Omega Optical, were used to cut off the excitation light and obtain high contrast fluorescence images. Additionally, a water-immersion objective lens (Nikon, Fluor 60X, NA1.0) is used for cell imaging.

### 2.7. Cell Line

Monocytic THP-1 cells (American Type Culture Collection [ATCC], Manassas, VA); were maintained at 37°C, under 5% CO_2_ in RPMI 1640 medium supplemented with 10% (v/v) heat-inactivated fetal bovine serum (FBS) (Sigma, St.Louis, MO), penicillin (100 units/mL), streptomycin (100 mg/mL), and L-glutamine (2 mM). THP-1 cells are cultured as a single-cell suspension cultures and were split at a ratio of 1 : 4 once a week.

### 2.8. HIV-1 Infection of THP-1

Undifferentiated THP-1 cells are susceptible to infection by T-tropic human immunodeficiency virus type 1 (HIV-1) isolates that use the coreceptor CXCR4 (X4 strains). Undifferentiated THP-1 (1 × 10^6^ cells/mL) were infected for 3 hours with HIV-1_IIIB_ (NIH AIDS Research and Reference Reagent Program) at a concentration of 10 ^3.0^TCID_50_/mL cells, equivalent to 10 ng viral isolate/mL of culture media. Following that, the infected cells were washed with Hanks buffered saline, reconstituted in RPMI media (fortified with 10% FBS) and incubated at 37°C/5% CO_2_ for 7 days. Levels of p24 in the culture supernatants were measured using a commercially available p24 ELISA kit (Zeptometrix, Buffalo, NY) 7 days postinfection. These infected THP-1 cells were then washed and reconstituted in fresh culture medium and used for evaluating the antiHIV-1 efficacy of the nanoplexes.

### 2.9. p24 ELISA

HIV-1 infection was monitored by the viral p24 level in harvested cell culture supernatants, using enzyme-linked immunosorbent assay (ELISA) plates obtained from a commercial vendor (Zeptometrix Buffalo, NY). The results are expressed in pg/mL as the mean and standard deviation (SD) of duplicate determinations from two wells.

### 2.10. Real-Time Quantitative PCR (Q-PCR)

Cytoplasmic RNA was extracted by an acid guanidinium-thiocyanate-phenol-chloroform method using Trizol reagent (Invitrogen, Carlsbad, CA) [16]. The amount of RNA was quantified using a Nano-Drop ND-1000 spectrophotometer (Nano-Drop, Wilmington, DE) and the isolated RNA was stored at −80°C until used. The LTR-R/U5 region represents early stages of reverse transcription of HIV-1. Following conversion of RNA to cDNA using reverse transcription, relative abundance of mRNA species was quantified by real-time quantitative PCR using the LTR/RU5-specific primers and the Brilliant SYBR green QPCR master mix (Stratagene Inc, La Jolla, CA; Cat# 600548-51). The followings are the primer sequences used for LTR/RU5 (Forward primer 5′-TCTCTCTGGTTAGACCAGATCTG-3′ and Reverse primer 5′-ACTGCTAGAGATTTTCCACACTG-3′). Relative expression of mRNA species was calculated using the comparative C_T_ method [17]. All data were controlled for quantity of RNA input by performing measurements on an endogenous reference gene, *β*-actin. In addition, results obtained on RNA from treated samples were normalized to results obtained on RNA from the control, untreated sample. Results were expressed as transcript accumulation index (TAI) [17, 18]. This calculation assumes that all PCR reactions are taking place with 100% efficiency.

## 3. Results

### 3.1. Characterization of the Nanoplexes

**Figure 1:**
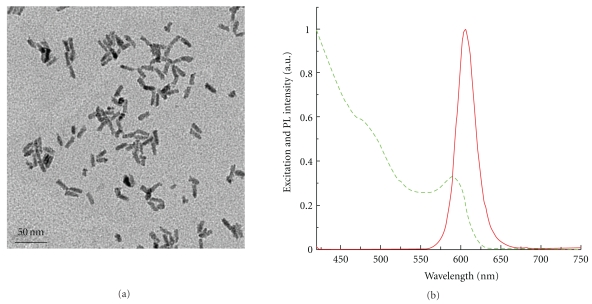
QR-si510 siRNA nanoplex characterization: Representative figures of (a) Transmission electron microscope image of nanoplexes showing that the average diameter of the nanoplex is ~15–20 nm (bar scale = 50 nm). No aggregation was observed after nanoplex formation. (b) Absorption (red line) and Emission spectra (green line) of QR-si510 siRNA nanoplexes.

The physical properties of the nanoparticle formulations are usually established using the following techniques: (a) dynamic light scattering to estimate the hydrodynamic diameter and surface charge of the aqueous dispersed nanoparticles, (b) transmission electron microscopy (TEM) to determine particle size and size distribution, and (c) evaluation of the spectral properties of the fluorescent nanomaterials using simple spectrophotometry and spectrofluorimetry.  Figure 1 shows the size-distribution of the QR-siRNA nanoplex via TEM and DLS measurements. TEM image (Figure 1(a)) shows the rods with average length and diameter of 15 nm.  Figure 1(b) shows the absorption and emission spectra of the free siRNA and the siRNA bound to the QR with the QR emission wavelength around ~620 nm. Transmission electron microscopy (TEM) images of nanoplexes show that they remain as individual particles with a diameter of ~15–20 nm (Figure 1(a)). It can be visualized from the TEM that siRNA complexation did not cause any aggregation of the QRs. The concentration of these positively charged QRs was approximately 0.1 mg/mL and the optimal concentration of QR that was used to conjugate with the si510 HIV-1 siRNA to form a nanoplex was 200 pg.

### 3.2. Effect of the QR-si510 HIV-1siRNA Nanoplex on Cell Viability in THP-1 Cells

**Figure 2:**
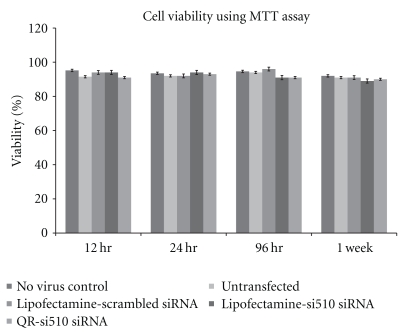
The time-dependent cytotoxicity of QR-si510 siRNA nanoplex on THP-1 cells using the MTT assay. Our results show absence of any toxicity in THP-1 monocytic cultures treated with lipofectamine transfected scrambled siRNA, Lipofectamine transfected si510 HIV-1 siRNA and the QR-si510 siRNA nanoplex as compared to the untransfected control over a 12 hr to 1 week time period. The concentration of both the scrambled siRNA and the si510 HIV-1 siRNA were 10 nM. Also included in the MTT assay were THP-1 cells not treated with the HIV-1 virus (no virus control). The results shown are mean ± SD of 3 separate experiments.

An MTT assay was done to evaluate the effect of the nanoplexes on cell viability of the THP-1 cells. Cell viability was also tested in the THP-1 cells prior to being infected by HIV-1 as well as after 7 days post infection and over 99% viability was observed in cells prior to be treated with the nanoplexes. THP-1 cell viability was measured after treatment with the nanoplexes at 12, 24, 96 hr, and upto 1 week posttransfection using the maximum dose (100 nM) of QR-si510 HIV-1 siRNA. Our results (Figure 2) show greater than 90% viability in THP-1 cells treated with QR-si510 HIV-1 siRNA nanoplexes and under all treatment conditions for up to 1 week. These results demonstrate that the QR-mediated delivery of siRNA did not produce any appreciable cytotoxicity in THP-1 cells.

### 3.3. Effect of the QR-si510 HIV-1siRNA Nanoplex on HIV-1 Replication

The ultimate goal of our studies was to evaluate the nanoplex uptake by the THP-1 cells and examine the efficacy of the nanoplex in inhibiting HIV-1 viral replication by measuring the antiviral activity in the cells and culture supernatants. To determine the nanoplex uptake by the THP-1 and the dose and time kinetics of the nanoplex THP-1 cells were treated with various concentrations of the si510 HIV-1siRNA ranging from 5 nM to 100 nM over a range of time periods from 6 hr to 1 week posttransfection and the antiviral efficacy the nanoplexes was tested. At the end of the incubation period, the THP-1 cells and culture supernatants were harvested and the antiviral activity was measured using the p24 ELISA assay, as well as gene expression of HIV-1 LTR was quantitated in the RNA extracted from the THP-1 cells using real-time PCR. 

**Figure 3:**
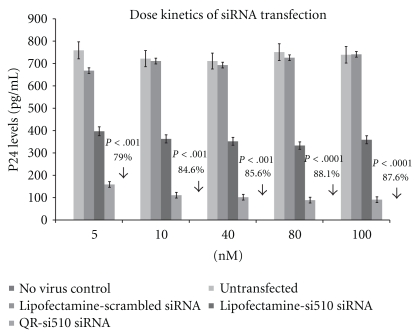
The dose-dependent kinetics of QR-si510 siRNA nanoplex on HIV-1-infected THP-1 cells. THP-1 monocytic cultures were treated with lipofectamine transfected scrambled siRNA (5–100 nM), Lipofectamine transfected si510 HIV-1 siRNA (5–100 nM) and the QR-si510 siRNA (5–100 nM) nanoplex for a 48 hr time period and the p24 production was measured in the supernatants at the end of the incubation period using a commercially available p24 ELISA kit. Untransfected HIV-1-infected THP-1 and the non-HIV-1-infected THP-1 cells (nonvirus control) were the experimental controls. The lipofectamine was used as per manufacturer's protocol. Our results show that 10 nM of the QR-si510 HIV-1siRNA nanoplex was optimal in achieving almost 85% suppression of viral replication. The results shown are mean ± SD of 3 separate experiments.

**Figure 4:**
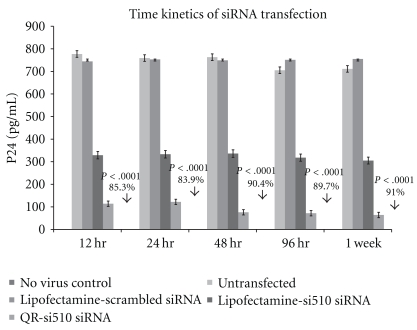
The dose-dependent kinetics of QR-si510 siRNA nanoplex on HIV-1-infected THP-1 cells. THP-1 monocytic cultures were treated with lipofectamine transfected scrambled siRNA (10 nM), Lipofectamine transfected si510 HIV-1 siRNA (10 nM) and the QR-si510 siRNA (10 nM) nanoplex for time period ranging from 12 hr to 1 week posttransfection. At the end of the incubation period, the p24 production was measured in the supernatants using a commercially available p24 ELISA kit. Untransfected HIV-1-infected THP-1 and the non-HIV-1-infected THP-1 cells (nonvirus control) were the experimental controls. The concentration of both the scrambled siRNA and the si510 HIV-1 siRNA were 10 nM and the lipofectamine was used as per manufacturer's protocol. Our results showed that 90% suppression of viral replication was observed 48 hr posttransfection and that further this suppression was observed even 1 week posttransfection. The results shown are mean ± SD of 3 separate experiments.

The results of our dose response experiments (Figure 3) show a significant decrease in HIV-1 p24 production in HIV-1-infected THP-1 cells transfected with the QR-si510 HIV-1siRNA nanoplexes as compared to HIV-1-infected THP-1 cells transfected with a commercially available transfection reagent Lipofectamine (Invitrogen, Carlsbad, CA). No significant differences in p24 levels was observed when THP-1 cells were treated with different concentration of si510 HIV-1siRNA which ranged from 5 nM to 100 nM. Comparisons between the untransfected control and the QR-si510 HIV-1 siRNA nanoplex transfected THP-1 cells show a significant decrease in p24 production. The p24 levels in cells treated with 5, 10, 40, 80, and 100 nM concentrations of si510 HIV-1 siRNA were 159 ± 9.6 pg/mL, 111 ± 10.9 pg/mL, 102 ± 11.3 pg/mL, 89 ± 8.3 pg/mL and 91 ± 9.6 pg/mL, respectively, that corresponded to a 79.05% decrease (*P* < .001), 84.63% decrease (*P* < .001), 85.65% decrease (*P* < .001), 88.15% decrease (*P* < .0001) and 87.69% decrease (*P* < .0001) in viral replication as measured by p24 production. While the concentrations of the si510 HIV-1 siRNA varies from 5–100 nM, the QR concentration in the nanoplexes was constant and was 200 pg. In our time kinetics experiments we used the optimal 10 nM concentration of si510 HIV-1 siRNA. Comparisons between the untransfected control and the QR-si510 HIV-1 siRNA nanoplex transfected THP-1 cells at 12, 24, 48, 96 and 1 week posttransfection showed a significant decrease in p24 production as early as 12 hrs posttransfection (Figure 4). The p24 levels in THP-1 cells at 12, 24, 48, 96 hr, and 1 week posttransfection were 114 ± 10.5 pg/mL, 122 ± 11.2 pg/mL, 76 ± 9.4 pg/mL, 72 ± 8.7 pg/mL and 64  ±  6.3 pg/mL, respectively, and corresponded to a 85.33% decrease (*P* < .0001), 83.93% decrease (*P* < .0001), 90.04% decrease (*P* < .0001), 89.79% decrease (*P* < .0001) and 91.00% decrease (*P* < .0001) in viral replication as measured by p24 production. The QR concentration in the nanoplex was 200 pg. The results of our time kinetics experiments show that 48 hrs posttransfection, a 90% suppression of viral replication was observed (Figure 3) and this suppression was observed even 1 week posttransfection. The 48 hr time point was determined to be the optimal time for transfection with the nanoplex, as a 90% decrease in p24 production was observed at that time point. 

**Figure 5:**
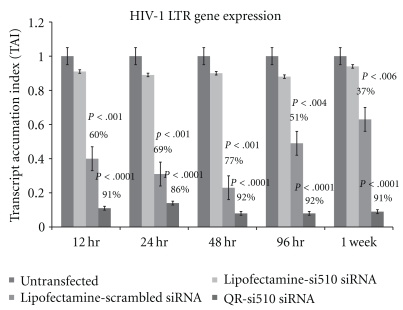
Effect of QR-si510 siRNA nanoplex on LTR/RU5 gene expression in HIV-1-infected THP-1 cells. THP-1 monocytic cultures were treated with lipofectamine transfected scrambled siRNA (10 nM), Lipofectamine transfected si510 HIV-1 siRNA (10 nM) and the QR-si510 siRNA (10 nM) nanoplex for time period ranging from 12 hr to 1 week posttransfection. At the end of the incubation period, RNA was extracted, reverse transcribed and the LTR/RU5 gene expression quantitated using Q-PCR. Our results show a significant decrease in HIV-1 LTR gene expression in HIV-1-infected THP-1 cells treated with the 10 nM QR-si510 HIV-1siRNA nanoplex at 12, 24, 48, 96 hr, and 1 week posttransfection as compared to the untransfected THP-1 cells and the scrambled control. The results shown are mean ± SD of 3 separate experiments done in duplicate.

Additional experiments to determine changes in viral replication by measuring HIV-1 LTR gene expression using real-time PCR were done using 10 nM concentration of the QR-si510 HIV-1 siRNA nanoplex at various time points ranging from 12 hr to 1 week posttransfection. The results of our HIV-1 LTR gene expression studies show a significant decrease in HIV-1 LTR gene expression in THP-1 cells treated with the 10 nM QR-si510 HIV-1siRNA nanoplex at 12, 24, 48, 96 hr, and 1 week posttransfection as compared to the untransfected THP-1 cells and also the scrambled control. The percentage decrease in HIV-1 LTR gene expression was 91% (TAI = 0.11 ± 0.02 versus 0.91 ± 0.05, (*P* < .0001)), 86% (TAI = 0.14 ± 0.06 versus 0.89 ± 0.03 (*P* < .0001)), 92% (TAI = 0.08 ± 0.03 versus 0.90 ± 0.07 (*P* < .0001)), 92% (TAI = 0.08 ± 0.05 versus 0.88 ± 0.03 (*P* < .0001)), and 91% (TAI = 0.09 ± 0.04 versus 0.94 ± 0.08 (*P* < .0001)) as compared to the scrambled control (Figure 5). The percentage decrease in HIV-1 LTR gene expression in THP-1 cells treated with the 10 nM of si510 HIV-1siRNA transfected with Lipofectamine at 12, 24, 48, 96 hr, and 1 week posttransfection were 60% (TAI = 0.40 ± 0.09 versus 0.91 ± 0.05, (*P* < .001)), 69% (TAI = 0.31 ± 0.07 versus 0.89 ± 0.03 (*P* < .001)), 77% (TAI = 0.23 ± 0.05 versus 0.90 ± 0.07 (*P* < .001)), 51% (TAI = 0.49 ± 0.11 versus 0.88 ± 0.03 (*P* < .004)), and 37% (TAI = 0.63 ± 0.06 versus 0.94 ± 0.08 (*P* < .006)), respectively, as compared to the scrambled control. These results confirm that the QR nanoparticles are a more effective than the commercially available transfection agent lipofectamine in not only delivering siRNA but also suppressing HIV-1 viral replication for a prolonged period of time.

### 3.4. Imaging of Nanoparticles

**Figure 6:**
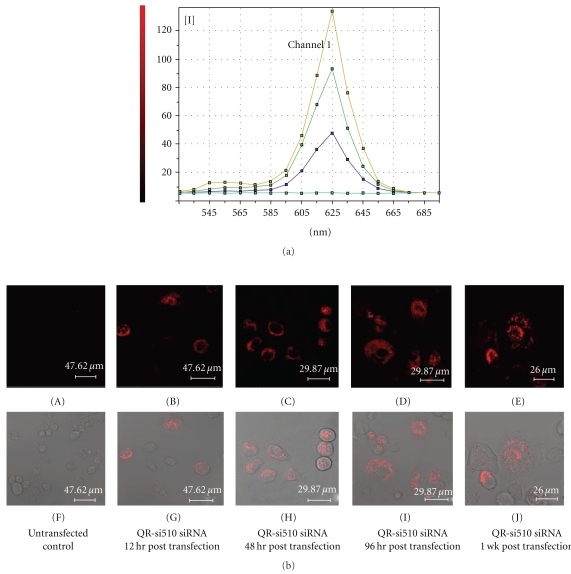
Confocal images of THP-1 cells treated QR-si510 siRNA nanoplexes: THP-1 cells were treated with QR-si510 siRNA nanoplexes for 12, 48, 96 hr, and up to 1 week and uptake of the nanoplexes was observed using confocal microscopy. (a) Representative local emission spectra confirms that the fluorescence signal in the THP-1 cells is localized to the QR si510 HIV-1siRNA nanoplexes, evident from the sharp emission spectra peak of 625 nm. (b) (A–J): Parallel Fluorescence and transmission images showing fluorescence (red) staining indicating QR-si510 siRNA nanoplex uptake by THP-1 cells.

The cellular uptake of the QR-siRNA nanoplexes was studied using confocal microscopy.  Figure 6(a) shows the confocal microscopic imaging and the local spectra of THP-1 cells treated with functional QR-si510 HIV-1siRNA nanoplexes. Confocal microscopy images were obtained with laser excitation at 405 nm.  Figure 6(a) shows the representative local spectra obtained from different points in the confocal images from cells. The red labeling of THP-1 cells confirms the fluorescence signal is localized to the QR si510 HIV-1siRNA nanoplexes which is evident from the sharp emission spectra peaking around 625 nm. Additionally, it is important to note that the cellular autofluorescence which is normally located around 500 nm shows no interference with the emission from QRs. Our results show uptake of the QR-si510 HIV-1siRNA nanoplexes by the THP-1 cells at 12, 48, 96 hr, and 1 week posttransfection (Figure 6(b) (A)–(J)).

### 3.5. Statistical Analysis

An Independent *t*-test or one-way ANOVA was applied to compare changes between transfected and untransfected groups. Comparison among the means was performed with the post hoc Bonferroni analysis test using the Prism statistical analysis software (GraphPad Software, Inc. La Jolla, CA). The quantitative data are presented as mean ± SD unless specified otherwise. Statistical significance was considered at *P* < .05.

## 4. Discussion

Developing strategies that treat HIV infection and eliminate persistent viral reservoirs are needed and gene therapy approaches such as RNA interference (RNAi) have emerged as a therapeutic possibility for the elimination of HIV-1 [19]. The RNAi pathway generates small interfering RNA (siRNA) from either long double stranded stretches of RNA or RNA hairpins, respectively, and this siRNA then guides an effector complex to a homologous sequence of mRNA and regulates suppression of gene expression. We choose an siRNA that targets the HIV-1 TAR element since the presence of TAR is important for activation of viral transcription by the HIV-1 Tat protein. Recently, Naito et al. did an exhaustive comprehensive computational analysis where they validated the functionality of several siRNA's that targeted multiple subtypes of HIV-1 and the highly conserved regions of the HIV-genome [7, 8]. They identified several highly effective siRNA's that have a potential to target >90% of HIV-1 strains, we choose an siRNA sequence from their list of highly effective siRNA's called si510 which targets the TAR/poly A region of the HIV-1 LTR. 

The use of semiconductor nanocrystals (NCs) or so-called quantum dots (QDs)/quantum rods (QRs) as luminescence probes for numerous biological and biomedical applications has become an area of intense research over the last decade [9–11, 20]. QRs offer several advantages over organic dyes, including increased brightness, stability against photobleaching, broad absorption spectra, and a tunable and narrow emission spectrum [9, 10]. As a result, they have demonstrated the potential to dramatically outperform conventional organic dyes in imaging of cellular and subcellular structures and in a variety of bioassays [9, 10, 13, 20]. More importantly, the surface of the QRs can be easily functionalized to incorporate biologicals such as siRNAs, antibodies, peptides, and proteins [20]. Furthermore, the size of these nanobioconjugates under physiological condition are typically less than 50 nm, making these QR-based nanoparticles ideal candidates for in vitro and in vivo studies and applications [14, 15, 21]. 

In this investigation, we evaluate the specificity and efficiency of QRs as nonviral gene nanocarriers for the si510 HIV-1 siRNA that targets the TAR/poly A region of the HIV-1 LTR thereby suppressing HIV-1 viral replication. We validated the process of complexing the si510 HIV-1 siRNA to a quantum rod (QR) and examined the efficacy, the dose and time kinetics of this QR-si510 HIV-1 siRNA nanoplex in suppressing viral replication in the HIV-1-infected monocytic cell line THP-1. 

Figure 1 showed the size-distribution of the QR-si510 HIV-1siRNA nanoplex via TEM and DLS measurements. Our TEM images of the nanoplexes show that the nanoplexes have a diameter of ~20 nm (Figure 1(a)) and that no aggregation of the QRs is observed demonstrating the colloidal stability of the nanoplex. The QR's we synthesized are positively charged and concentration of these positively charged QRs was approximately 0.1 mg/mL. The concentration of QR in the QR-si510 HIV-1 siRNA nanoplex was 200pg; however, the concentration of the siRNA within the nanoplex ranged from 5 nM to 100 nM. Our data suggest that as little as 10 nM quantity of the QR-si510 HIV-1siRNA nanoplex was optimal in achieving almost 85% suppression of viral replication (Figure 3). 

The optimal amount of QR available for complexation with the siRNA was determined based on our previous studies using QR that were synthesized based on similar methodologies and had similar charged and concentration as determined by their Optical density (OD) measurements [14, 15, 21]. The results of our time kinetics experiments showed that 90% suppression of viral replication was observed 48 hr posttransfection and further this suppression was observed even 1 week posttransfection (Figure 4). Comparisons between lipofectamine and the QR's indicate that the QR's are superior to lipofectamine in not only delivering siRNA thereby suppressing HIV-1 viral replication as indicated by the significant decrease in p24 levels and by the significant decrease in HIV-1 LTR gene expression (Figure 5), but also that they are able to prolong the viral suppression for a extended period of time possibly due to the slow release of siRNA into the cells. Further, our imaging studies (Figure 6) show significant uptake of the QR-si510 HIV-1siRNA nanoplexes by the THP-1 and localization of these nanoplex within the cell was observed even 1 week posttransfection. Additionally, it is important to emphasize that no significant cell toxicity was observed when the THP-1 cells were treated with the nanoplexes and >95% viability was observed even 1 week posttreatment (Figure 2). 

Gene therapy techniques such as siRNA possess great potential in HIV-1 therapeutics; however, they must overcome the key obstacle of reaching the interior of the HIV-1-infected cells. Nanoparticles have the potential to be a successful delivery vector for antiHIV-1 siRNA, anti-HIV drugs since these nanoparticles can be modified to improve the stability of the drug/genetic material in the biological environment; and have the capacity to mediate the biodistribution of active compounds; the ability to load, transport, target, and release their therapeutic payloads and can facilitate transport of these therapeutic payloads across the blood-brain barrier (BBB). Thus, the QR-si510 HIV-1 siRNA nanoplexes provide an advantage over current therapeutic options, with respect to delivery and release of genetic material to a specific cell. These studies indicate that the nanoplex consisting of QR-si510 HIV-1 siRNA is both safe and effective in suppressing viral replication, thus confirming that nanotechnology will play a significant role in future HIV-1 therapeutics.
